# Risk factors among people surviving out-of-hospital cardiac arrest and their thoughts about what lifestyle means to them: a mixed methods study

**DOI:** 10.1186/1471-2261-13-62

**Published:** 2013-08-27

**Authors:** Ann-Sofie Forslund, Dan Lundblad, Jan-Håkan Jansson, Karin Zingmark, Siv Söderberg

**Affiliations:** 1 Department of Research, Norrbotten County Council, Luleå SE-971 89, Sweden; 2 Department of Health Science, Division of Nursing, Luleå University of Technology, Luleå SE-971 87, Sweden; 3 Department of Medicine, Sunderby Hospital, Luleå SE-971 80, Sweden; 4 Department of Public Health and Clinical Medicine, University of Umeå, Umeå SE-901 87, Sweden; 5 Department of Medicine, Skellefteå Hospital, Skellefteå SE-931 41, Sweden

## Abstract

**Background:**

The known risk factors for coronary heart disease among people prior suffering an out-of-hospital cardiac arrest with validated myocardial infarction aetiology and their thoughts about what lifestyle means to them after surviving have rarely been described. Therefore the aim of the study was to describe risk factors and lifestyle among survivors.

**Methods:**

An explanatory mixed methods design was used. All people registered in the Northern Sweden MONICA myocardial registry between the year 1989 to 2007 who survived out-of-hospital cardiac arrest with validated myocardial infarction aetiology and were alive at the 28th day after the onset of symptoms (n = 71) were included in the quantitative analysis. Thirteen of them participated in interviews conducted in 2011 and analysed via a qualitative manifest content analysis.

**Results:**

About 60% of the people had no history of ischemic heart disease before the out-of-hospital cardiac arrest, but 20% had three cardiovascular risk factors (i.e., hypertension, diabetes mellitus, total cholesterol of more or equal 5 mmol/l or taking lipid lowering medication, and current smoker). Three categories (i.e., significance of lifestyle, modifying the lifestyle to the new life situation and a changed view on life) and seven sub-categories emerged from the qualitative analysis.

**Conclusions:**

For many people out-of-hospital cardiac arrest was the first symptom of coronary heart disease. Interview participants were well informed about their cardiovascular risk factors and the benefits of risk factor treatment. In spite of that, some chose to ignore this knowledge to some extent and preferred to live a “good life”, where risk factor treatment played a minor part. The importance of the support of family members in terms of feeling happy and having fun was highlighted by the interview participants and expressed as being the meaning of lifestyle. Perhaps the person with illness together with health care workers should focus more on the meaningful and joyful things in life and try to adopt healthy behaviours linked to these things.

## Background

Several cardiovascular risk factors have been identified since the 1960s. The first causal factors recognized were hypertension, hypercholesterolemia, and tobacco use; thereafter, other novel risk factors associated with psycho-social surroundings and behaviour have been added [1,2]. Psycho-social risk factors are associated with for example peoples socio-economic status, emotions like anxiety and depression and work overload [3]. According to the World Health Organization (WHO), behavioural risk factors cause about 80% of cardiovascular disease (CVD) in the world. Such risk factors include an unhealthy diet, physical inactivity, obesity, and tobacco use [4]. Despite this knowledge, not all people with coronary heart disease (CHD) get evidence-based treatments; or if they do, they often do not reach the guideline goals [5-7], thereby leading to risks of complications and premature death [8-10]. For some people, an out-of-hospital cardiac arrest (OHCA) might be the first symptom of CHD [11-15]. The incidence of treated OHCA varies between 28–55 per 100 000 inhabitants yearly and the overall survival to discharge is low 2–11% [16].

For people with coronary heart disease (CHD) secondary preventive measures including changes in behavioural risk factors and lifestyle are important to ensure future health and prevent complications [17-21]. However, studies have indicated that lifestyle changes are difficult to maintain [22] and support for people making lifestyle changes is crucial [23,24]. To the best of our knowledge, no studies have described which known risk factors for CHD people had before they suffered out-of-hospital cardiac arrest with validated myocardial infarction aetiology (OHCA-V). Furthermore no studies have described survivors’ thoughts about what lifestyle means to them. This knowledge can be used in primary preventive care giving health care personnel information about risk factors among people suffering OHCA-V. It should also provide a deeper understanding of surviving people’s own thoughts about risk factors associated with their lifestyle which could be used to identify ways in which to help people in a more supportive and individually suited way in both primary preventive care and in cardiac rehabilitation. With a mixed methods design this study present known risk factors among people before OHCA-V and what lifestyle means to them after surviving.

## Methods

### Design

An explanatory mixed methods design with a participant selection model was used [25].

### Setting

Multinational MONItoring of trends and determinants in CArdiovascular disease (MONICA) is a WHO initiated project intended to monitor trends in cardiovascular disease and is on-going in northern Sweden since 1985. From the beginning people aged 25 to 64 were included but since the year 2000 the inclusion criteria are increased to 74 years of age [26-28].

The Västerbotten intervention programme (VIP) is a community intervention programme intended to reduce morbidity and mortality from CVD and diabetes in the county of Västerbotten, Sweden. In this programme, people aged 40, 50, and 60 have been invited to participate in individual counselling about healthy lifestyle habits and screening for risk factors [29].

### Sample/participants

In the first phase quantitative data were selected from the Northern Sweden MONICA project. Included are all people who resided in the area of Norrbotten and Västerbotten who suffered an OHCA-V, were alive at the 28th day after the onset of symptoms, and were registered in the Northern Sweden MONICA project between 1989–2007 (n = 71). In the second phase participants in the quantitative phase were used to guide purposeful sampling for a follow-up in depth qualitative study. All people still alive on the 25th of January in 2011 (n = 46) were sent a personal letter that explained the aim of the study and asked for their participation, i.e., completion of a questionnaire focusing on their risk factors for CHD before suffering OHCA-V and to participate in an interview on what lifestyle means to them after surviving. The risk factors registered in the MONICA project and VIP were about the same as in the questionnaire. Thirty-two people answered the questionnaire and of those 15 chose to participate in the interviews. The first author phoned those who had decided to participate and made an appointment for their individual interview. Two of the participants decided not to participate when time for the interview was scheduled. The interview participants all suffered their OHCA-V 4 to 17 years prior to the interview (md = 8 years) and were 52 to 81 years of age (md = 68 years) at the time of the interview.

### Data collection

Participants’ (n = 71) sex, marital status, work status, and coexisting conditions like ischemic heart disease (IHD), myocardial infarction (MI), hypertension, diabetes mellitus (DM), and smoking habits prior suffering the cardiac arrest, were derived from the Northern Sweden MONICA project, registered at the participants’ OHCA-V event. This information was compiled with data prior the OHCA-V from the VIP, which also contributed data on total cholesterol and body mass index (BMI). Answers from the questionnaire were used to add to data about their risk factors. Where data regarding the risk factors was insufficient, an additional medical journal review was conducted to make the data as complete as possible; yet, insufficient data still ranged between 2.8 and 5.6% for marital and work status and 5.6 and 11.3% for total cholesterol, smoking habits, and BMI. Nevertheless, the data for the history of other coexisting conditions was sufficient. Measurements regarding total cholesterol and BMI were from before or in association with the OHCA-V event. Cholesterol measurements were divided into two groups: i.e., ≤ 4.99 mmol/l and ≥ 5.0 mmol/l and/or taking lipid lowering medication. Moreover, BMI values were divided into three groups: i.e., ≤ 24.99 kg/m^2^, 25–29.99 kg/m^2^, and ≥ 30 kg/m^2^[30].

### Interviews

Personal interviews were conducted in 2011 with the participants (n = 13). The qualitative interviews were performed as conversations [31] focusing on the following questions: “Please tell me what you think when I say “lifestyle”? What does lifestyle mean to you? Has your cardiac arrest influenced your lifestyle? What is important to make you feel good?” Clarifying questions were asked, e.g., Can you tell me more? What do you mean? Can you give an example? All participants chose to be interviewed in their homes. Each interview lasted about an hour (md = 55 min), was tape recorded, and later transcribed verbatim.

### Ethical considerations

The study was approved by the Regional Ethical Review Board. The participants in the interview study gave their informed consent and were assured confidentiality and an anonymous presentation of the findings.

### Data analysis

The characteristics of the participants are presented as absolute numbers and proportions. The text from the interviews was analysed with a qualitative manifest content analysis [32]. The interview text was read several times while considering the aim of the study in order to obtain a sense of the content. In the next step of the analysis, textual units were identified, extracted, and sorted into content areas describing a specific, explicit area. In each content area, a category was created to summarize the content. Finally, the categories were divided into subcategories.

### Validity and reliability/Rigour

The quantitative data included are collected through strictly validated procedures (25–27, 29) which also strengthen reliability. Rigour of the qualitative analysis was established through repeated discussions between the first and last author, and finally all authors came to an agreement about the results. The categories are validated with quotations from the interview texts. Findings from this study might be transferred to similar settings, for example people suffering acute illness and thinking about what lifestyle means. The qualitative component in the study adheres to RATS guidelines on qualitative research.

## Results

The results are presented in two parts. Part I presents the results of the quantitative analysis, and Part II presents the results of the qualitative analysis.

### Part I: Risk factors prior OHCA-V among surviving people

The results (Table 1) show that most people were married or cohabitated and 60% were gainfully employed when they suffered OHCA-V. Sixty percent had no prior history of IHD or hypertension, whereas 25% and 17% had been diagnosed with MI and DM, respectively, prior the OHCA-V. Eighty percent of the people had total cholesterol levels greater than 5.0 mmol/l and/or lipid lowering medications. Almost half were smokers and 63% were overweight/obese. The characteristics of the interview participants compared to the whole group of participants showed a higher proportion of people that smoked, were overweight, had total cholesterol > 5 mmol/l and/or lipid lowering medication but only one were diagnosed with DM.

**Table 1:** **Characteristics prior onset of OHCA**-**V for people alive 28 days after the OHCA**-**V** (**n** = **71**) **and for interview participants** (**n** = **13**)

	**People alive 28 days after the OHCA**-**V (n** **=** **71)**		Interview participants* (n **=** **13)**	
	**n**	**%**	**n**	**%**
**Sex**, male/female	53/18	74.6/25.4	10/3	76.9/23.1
**Age**	58.3 (9.1)		57.5 (9.0)	
(years, mean ± SD, min-max)	35-74		43-75	
**Marital status**				
married/cohabitant	58	84.1	11	84.6
**Work status**				
gainful work	41	61.2	8	61.5
**History of coexisting conditions**				
IHD	28	39.4	4	30.8
previous MI	18	25.4	4	30.8
hypertension	28	39.4	4	30.8
DM	12	16.9	1	7.7
total cholesterol, mmol/L				
≤ 4.99	14	20.9	1	7.7
≥ 5.0 and/or lipid lowering medication	53	79.1	12	92.3
**Smoking habit**				
smoker	31	47.7	8	61.5
former smoker	17	26.2	1	7.7
never smoked	17	26.2	4	30.8
**BMI**, **kg**/**m**^**2**^				
< 24,99	23	36.5	3	23.1
25-29,99	30	47.6	8	61.5
30-39,99	10	15.9	2	15.4
≥ 3 risk factors**	16	22.5	3	23.1

*IHD* ischemic heart disease, *MI*myocardial infarction, *DM* diabetes mellitus, *BMI* body mass index, * Interview participants are also included in People alive 28 days after the OHCA-V, ** hypertension, DM, total cholesterol ≥ 5.0 and/or lipid lowering medication, smoker.

### Part II: Peoples thoughts about what lifestyle means to them after surviving OHCA-V

The analysis revealed three categories and seven sub-categories (Table 2). The quotations is referenced to the participants, male = M and female = F.

**Table 2:** **Overview of categories and sub**-**categories that emerged from the analysis of the interviews with people who survived OHCA**-**V** (**n** = **13**)

**Categories**	**Sub**-**categories**
Significance of lifestyle	Finding joy and strength in meaningful relationships
	Feeling well and doing things of their choice
Modifying the lifestyle to the new life situation	Finding a reason why it happened and making lifestyle changes
	Making your own assessment of a risk behaviour
A changed view on life	Feeling grateful for a second chance at life
	Finding motivation for lifestyle changes and wishing to influence family members to adopt lifestyle changes
	Challenging one’s fears and adopting a positive outlook on life

### Significance of lifestyle

#### ***Finding joy and strength in meaningful relationships***

The participants described that after the OHCA-V, their lifestyle focused mainly on the importance of having people around. Relationships with their partners, children, grandchildren, siblings, and friends were important and a source of happiness and strength. Social interaction and fellowship included feelings of being needed and meaning something to others. Most of the participants were married or cohabited with others, and they talked about family as their most important relationships. Their partner was described as their companion in life, the one with whom to share things, and the one who cared and looked out for them. Children and grandchildren were given high priority because spending time with them, attending their activities, inviting them to dinner, and being able to help with babysitting were identified as true sources of joy. Participants talked about the importance of having fun and laughing with others.

We get to be there in the mountains with our grandchildren. They are skiing, and I can sit there in the snow, feeling great. Seeing them is so much fun: that you get to be with them, that is life-joy (F2).

#### ***Feeling well and doing things of their choice***

The participants described that after surviving OHCA-V they did not take feeling well for granted. They described that their lifestyle was connected with feeling well. Participants talked about doing things they had a desire to do and found pleasure in doing. The most important thing identified by all was being able to occupy and engage themselves in something they found meaningful. Overall, the participants’ thoughts were imbued by what was good for them.

… do stuff that you feel a desire of doing; don’t say ‘no’. Take the opportunity and don’t think too much, but do it. Indulge yourself in doing these things (M12).

Several of the participants talked about how exercising made them feel well; they had different abilities to exercise according to their medical status after the OHCA-V. Some participants went to the gym, went skiing, and played tennis, while others were pleased when they were able to go out for a walk, go to the public swimming pool, or attend senior citizen dances. All tried to find a way to exercise, and for some participants medications helped them to be able to do physical activities. Those who lived in a house had housework that they enjoyed, such as carpentry, mowing the lawn, shovelling snow, even when they had to ask for help with some physically demanding tasks.

It’s my health promotion to shovel snow and mow the lawn. I think it is fun. I don’t need to shovel like this because that I’m not able to do, but to push like this is ok, and I can stop when I’m out of breath (M3).

Some of the participants felt good when visiting the forest or the archipelago to hunt or fish. Several participants were involved in an association and attended different events, and some enjoyed travelling. Three people still had gainful employment and found joy in working and meeting other people at work. All participants talked about the fact that food had been given more attention after their OHCA-V. They thought about what kind of food was good for them and balanced that with thoughts about how life should be good and they should feel well. That meant they sometimes ate food and snacks that they knew were not the best for them, but they believed that life should be enjoyed. All participants also talked about food in relation to their weight.

Before this event I was eating everything. I didn’t care what I ate; I ate everything that was good. Now I think about it (M6).

### Modifying the lifestyle to the new life situation

#### ***Finding a reason why it happened and making lifestyle changes***

After surviving an OHCA-V, the participants began to consider the reason for the MI, and according to the reasons identified, they tried to make appropriate lifestyle changes. Smoking cigarettes was one reason discussed. Of the eight people who smoked when they suffered their OHCA-V, five of them stopped at the OHCA-V event. All talked about the effect of heritage on CHD as a reason for their problems, and they indicated that they could do nothing about this. One participant said, “Nobody smoked in my father’s family, still they all had an MI” (F8). Three participants continued smoking by choice.

All participants talked about the effects of working and life being filled with negative stress. Several had been in leading positions in their jobs with responsibilities for staff, and some had been self-employed. The stress included long working hours and mental and economic anxiety. Participants expressed that they were not really surprised that the OHCA-V happened because they knew that their life situation prior to the OHCA-V was not sustainable. They talked about physical symptoms related to negative stress, like headaches, migraines, and body pain. However, even if they had understood the connection, they did not have the ability to make a change at the time.

I was working like a fool. I had three jobs at the same time, and there were times when I felt the adrenalin levels up in the hairline. So afterwards, I was not exactly surprised; I had simply run out of myself. Even if I knew it was wrong, I was not capable to do anything about it (M12).

They needed to survive the OHCA-V in order to clearly see their own working situation and decide what to do in the future. Some expressed gratitude for the event and viewed it as a turning point that was needed for making necessary changes.

The nurse said, I hope you understand that your life will not be the same. I looked at her and smiled and said, Thank God for that. Because what I had I would definitely not want back; I had already decided then to stop with the gasoline station, because it was the reason for the MI. It had dragged me down completely with negative stress for about 15 years (M 10).

Some participants made radical changes in their work life, and for some, the changes were more or less forced on them because of their changed physical capacity after the cardiac arrest. The event led to an awareness of tasks and situations related to stress and a changed approach when deciding what to or what not do. They made a stand and tried to avoid stress as much as they could, and they expressed that this was a positive change.

What I don’t have time to do today, I can do tomorrow. It doesn’t matter if it’s dust in the corner, I’ve to live with it, I’ll do it when there is time (F13).

#### ***Making your own assessment of a risk behaviour***

Some participants expressed that they were focused on being fit and maintaining a normal weight, but others seemed to be less focused on that. Participants said that they were aware of the need to exercise more, lose weight to get to a healthy weight, and eat according to all the rules. However, they felt that this was a choice to make and balance with the importance of feeling well and enjoying all things in life.

If I did it by the books – eat right, exercise right – then I probably would feel good but I feel good anyway doing my lifestyle (F13).

As previously mentioned, three people still smoked cigarettes after the OHCA-V, and they had each made an assessment of the risks related to smoking. One participant said he smoked so little that it did not matter. Two people said the cigarettes calmed them down to an extent. One woman said that she sorted through the advice she was given and concluded, “They can say what they want, but I still do as I want, and I alone take the consequences” (F8). One participant said perhaps 95% of her wanted to quit smoking, but the other 5% considered cigarettes to be a comfort when she was feeling down; the latter attitude actually outweighed the former in terms of her behaviour. Another participant had several relatives and friends that had stopped smoking and still developed lung and brain cancer; therefore, he said, “The physician asked me if I would quit smoking, and I said yes; when I die, I will stop automatically” (M11).

### A changed view on life

#### ***Feeling grateful for a second chance at life***

Most of the participants expressed thankfulness for surviving. Many thought that their survival was a miracle because everything had worked out so well when they suffered their OHCA-V. Indeed, they often described coincidences in their favour, such as the right people being around at exactly the right time. They told stories about the day on which they had their OHCA-V that included ordinary people knowing CPR and people from the fire department and ambulance being around when it happened; all of these circumstances highly contributed to their survival.

But I feel every morning when I wake up, I get one more day….. it’s a bit like being lucky or unlucky or coincidences (M12).

Some participants expressed that after their OHCA-V they were more easily moved and cried more often. They felt truly lucky, and several people basically stated, “It was not meant for me to die; it was meant for me to continue living” (M1).

#### ***Finding motivation for lifestyle changes and wishing to influence family members to adopt lifestyle changes***

Participants expressed that the OHCA-V was a wake-up call, and they stated that they did not want their life to be over. They wanted to continue to be with their partner in life and to see their children and grandchildren grow up. This attitude motivated lifestyle changes in order to increase their chances for a continued life.

I was afraid I wouldn’t be allowed to continue living. I’ve been given a second chance, and I will not aggravate it with cigarettes. If I start smoking again, there is a greater risk that I will get another MI, and to calm down, not stress so I don’t get another MI. This I’ve had to re-learn (F13).

The participants have all told their children of their family history with CHD and told them of the importance of getting regular healthy checks and informing healthcare personnel that they have a family history of CHD. They also recognized the effects of genetics when their children also developed high levels of cholesterol and high blood pressure and needed medical treatment.

I’ve preached to my daughters to check your blood pressure and cholesterol (F8).

Two participants that smoked had children that had tried to quit smoking but failed. The participants did not express any distress about this. In fact, one said, “the younger one tried to quit. She said, ‘No I cannot do it’. She was so nervous and angry and everything, so I told her to start smoking again” (M11).

#### ***Challenging one*’*s fears and adopting a positive outlook on life***

Participants said that after their cardiac arrest they were afraid of doing certain things such as solitary activities or physical activities; moreover, they said that they had challenged those fears. They said that they could not walk around being afraid of doing things, having another MI, or dying. They decided that they should not waste time worrying over something that was about to happen because they could do nothing about it if it occurred anyway.

I couldn’t go out alone, my life would be ruined if I didn’t have my freedom. Finally, I forced myself out into the woods, and there was a lot of agony in that decision, but I thought if I’m about to die, it doesn’t matter where I am and how it happens. I turned off my mobile phone, and sat down. Nobody knows where I am, and I feel damn good. After sitting there for a while philosophizing. I kept on walking, and then I experienced in a way that. I had broken the anxiety a bit. After that, I’ve been out many times by myself (M10).

Participants described trying to be as positive as possible and maintaining a happy attitude about things. Laughing and having fun was described as important, powerful aspects in life. They also described the positive aspects of trying not to worry, but instead first looking at situations as opportunities rather than difficulties and problems.

## Discussion

In this study, an explanatory mixed methods design was used to describe which known risk factors for CHD people had before they suffered out-of-hospital cardiac arrest and their thoughts about what lifestyle means to them after surviving. The aetiology of the OHCA in our study was validated MI where 40% had history of IHD but 60% had not. This means some people suffering OHCA-V are unaware of having an atherosclerosis disease and are not known in health care and available for preventive measures. A study [33] including people who were 25 to 64 years old with a first MI but no OHCA in the same region in northern Sweden found that a lower proportion of their participants had a history of IHD, hypertension, and DM before the event, as compared to those in our study who had suffered OHCA-V. People suffering OHCA-V were not free from risk factors but as many as 20% had three cardiovascular risk factors. This fact points to the importance of both primary and secondary preventive measures in the effort to avoid CVD and possible associated complications, including OHCA-V [34].

In our study most people were married or cohabited, and all interviewed participants talked in a loving manner about their partner when talking about lifestyle. This might have led to a better prognosis since feelings of high social support are health promoting [35]. Supportive social relationships impact health outcomes and are important for adjustment to illness for people with heart failure. Social support is explained as influencing health outcomes by helping the individual to look at stressful happenings in a less stressful way, and social support is implicated with healthy behaviours, like not smoking, adequate food intake, and healthy exercise habits [36]. Meaningful relationships have also been found to be important for women’s well-being after an MI. Close relationships provide strength, happiness and joy in life [37].

All participants talked about the negative effects of stress at work and believed that stress at work contributed to their OHCA-V. They had tried to make changes regarding stress at work and stress in general after the OHCA-V, but notably, none of the participants talked about getting help from health care professionals with stress management. Psychosocial factors, like stress at work, can affect people’s health. Job strain, low job control, and effort-reward imbalance has been studied and linked to adverse cardiovascular effects [38]. Psychosocial stressors, i.e., stress at work, stress at home, financial stress, and major life events in the past year, were shown to be associated with an increased risk of MI. The effects were found to be similar in both sexes, at various ages, and in different geographic regions of the world [39]. This might indicate that primary prevention should pay more attention to stress levels and that cardiac rehabilitation should involve stress management to a larger extent.

In the present study, the participants emphasized the importance of having people around for whom they cared and who cared for them. They were grateful for still being alive and were determined to keep on living to get to be with significant people in their surroundings. This emotion also gave them motivation to change behaviours. They focused on trying to do things that were enjoyable, having fun, and balancing these enjoyable activities with things that they knew they should do, like health promoting choices. This can be compared to the results of a study exploring patients’ perspectives on making and maintaining lifestyle changes after MI [40]. In that study, patients expressed that they were aware of harmful behaviours and were not really surprised that they were affected by a MI because they knew they had bad habits. They tried to make changes, but making changes was found to be more difficult than they had thought. Survival was a motivator for making lifestyle changes and they felt they had been lucky and expressed gratitude for being alive. Stress also emerged as a contributing factor for MI. A study investigating women’s knowledge of cardiovascular risk factors, self-care, and healthy behaviours showed that women’s knowledge was not significantly related to heart-healthy behaviours [41]. Indeed, a gap existed between knowing what to do and implementing the recommended behavioural changes. The intention to act and make changes did not translate into action.

This study has limitations. Although this study included all people who had survived an OHCA-V during a 19 year period in northern Sweden, this population was small. The MONICA project’s limitation in age inclusion means that people younger than 25 and older than 75 are not included in our study. The number of interviews performed was determined by the number of people who chose to participate.

## Conclusions

The results of this study reveal a paradox. The results from the interviews show that the participants seem to be well informed about their cardiovascular risk factors and the benefits of risk factor treatment and behavioural changes. In spite of that, some chose to ignore this knowledge at least to some extent and preferred living a “good life”, where risk factor treatment played a minor part. This is important knowledge and either implies that information about risks is still insufficient or simply that they value their life choices and take responsibility for the consequences of their decisions.

Still, the results of this study show the importance of primary prevention making people aware of cardiovascular risk factors and identifying unhealthy behaviours so that necessary lifestyle changes can be initiated before the onset of CHD. The challenge is to make people aware although they feel healthy and do not feel any symptoms of illness. In cardiac rehabilitation, health care personnel should try to get a better understanding of each person’s life situation and adjust preventive measures to fit the patient’s life situation. However, the following question must be raised: how can people get motivated to change unhealthy behaviours if they believe that they already have a good life? Perhaps another paradox lies in the preventive work: i.e., health care professionals should support people in their decisions about their chosen lifestyle so that people improve their quality of life as much as possible. The importance of the support of family members in terms of feeling happy and having fun was highlighted by the interview participants and expressed as being the meaning of lifestyle. Perhaps the person with illness together with health care workers should focus more on the meaningful and joyful things in life and try to adopt healthy behaviours and lifestyle changes linked to these things. Perhaps a combination of factors increasing the patient’s well-being can be the core to a good life despite illness.

## Competing interests

The authors declare that they have no competing interests.

## Authors’ contributions

All authors participated in the design of the study and worked with data analysis. ASF performed data collection, the major part of the data analysis and drafted the manuscript. KZ and SS drafted the manuscript. All authors read and approved the final manuscript.

## Pre-publication history

The pre-publication history for this paper can be accessed here:

http://www.biomedcentral.com/1471-2261/13/62/prepub
